# Impact of Vascular Service Centralization on the Carotid Endarterectomy Pathway: A Study at the Bedfordshire, Luton, and Milton Keynes Vascular Network

**DOI:** 10.7759/cureus.49726

**Published:** 2023-11-30

**Authors:** Abdul Hakeem, Mojahid Najem

**Affiliations:** 1 Vascular Surgery, Bedfordshire-Milton Keynes Vascular Centre, Bedford, GBR

**Keywords:** centralization, carotid endarterectomy (cea), carotid artery stenosis, transient ischemic attacks, hub and spoke

## Abstract

Introduction

Carotid endarterectomy (CEA) is the gold standard intervention for patients experiencing transient ischemic attacks (TIAs) or embolic strokes with >50% internal carotid artery (ICA) stenosis supplying index hemispheric territory. The recommended period for CEA is 14 days post-index event; this period carries a heightened risk for second ischemic events. However, implementation of this stringent timeline often encounters delays stemming from multifaceted factors. The centralization of vascular services, designed to enhance patient care, introduces a paradigm shift. Centralization's efficacy in improving patient outcomes, particularly in the CEA pathway, is a subject of ongoing investigation. Our study aims to discern the impact of centralized services on the timeliness of CEA for symptomatic carotid artery stenosis, shedding light on this complex interplay of factors.

Methods

This retrospective study analyzed CEA data at the Bedfordshire, Luton, and Milton Keynes Vascular Network between January 2021 and June 2023. Eligible patients exhibited symptomatic carotid artery stenosis, with asymptomatic cases; those unfit for surgery or receiving best medical therapy only were excluded. Patients were categorized by their primary referral location: Hub, Spoke-1, or Spoke-2. Demographic and referral data were collected, and timelines from symptom onset to surgery were recorded. Continuous variables were expressed as means and standard deviations, and categorical variables as counts and percentages. Box plots illustrated the relationship between referral origin and surgery timing, and the Classification and Regression Tree (CART) assessed second events. Statistical significance was determined using Fisher's exact and chi-square tests, with p<0.05 indicating significance.

Results

A total of 148 patients underwent CEA after implementing exclusion criteria. 35.5% (n=53) of patients were referred from the Hub, while 45.6% (n=67) and 18.8% (n=28) were from Spoke-1 and Spoke-2, respectively. 40% (n=59) received CEA within the recommended timeframe, and 15.4% (n=23) experienced a second ischemic event pre-surgery. Time from TIA clinic review to referral was 5.5±8 days and 16.4±20 days from vascular referral to surgery. Patterns of delays were observed, with Spoke-2 exhibiting the most significant delays. Notably, amaurosis fugax and embolic stroke correlated with recurrent ischemic events, emphasizing the importance of timely care in CEA.

Conclusion

Our study underscores the significant benefits and challenges of the Hub and Spoke model in vascular surgery. The growing referral delays from Spoke sites are concerning, emphasizing the need for a multi-disciplinary team approach within Spoke sites to ensure efficient and standardized care delivery.

## Introduction

Carotid endarterectomy (CEA) is the gold standard intervention for patients who have experienced an ischemic cerebrovascular event (transient ischemic attacks (TIAs) or embolic strokes) secondary to >50% internal carotid artery (ICA) stenosis supplying index hemispheric territory [1-4]. CEA offers maximum benefit when performed within a therapeutic window of 14 days post-index event; various guidelines have adopted the evidence to recommend surgery within 14 days [5-8]. The literature has increasingly underscored the profound implications of the immediate post-event period, revealing that the risk of a second ischemic event is considerably higher within the initial days following the index cerebrovascular event [1,5,9].

Despite the imperative nature of timely intervention, delays in practice are encountered. These delays result from a complex interplay of multifaceted factors, ranging from the speed of patient referral to prolonged waiting times for access to operating theatres [1]. The quest for enhanced patient care and optimized outcomes has led to centralizing vascular services, a strategy extensively explored in contemporary vascular literature [10].

Centralization in vascular healthcare represents a fundamental and transformative paradigm shift, introducing a reconfiguration of services to optimize patient care, streamline resource allocation, and enhance clinical outcomes. This care delivery model reimagines vascular services as being accessible round-the-clock, with a focus on high-volume facilities acting as central "Hubs" supported by regional "Spoke" sites. The overarching objective of centralization is to deliver improved care, emphasizing efficiency, quality, and timely access to interventions [11]. The efficacy of centralization in yielding enhanced patient outcomes is a burgeoning interest within the domain of vascular surgery. While considerable attention has been directed toward aneurysm surgeries, where centralization has yielded demonstrable benefits, the impact of centralization on the CEA pathway remains a subject of inquiry [10]. This critical juncture is underscored by the divergence in findings across various studies, where certain studies postulate that centralization yields marginal benefits when juxtaposed with the pre-centralization era [12].

Centralization's intricate tapestry reveals paradoxes, particularly in its influence on referral times and morbidity rates in cases of acute vascular issues [13]. While centralization has demonstrated commendable outcomes in specific contexts, it may inadvertently elongate the period between referral and intervention, culminating in heightened morbidity rates [10,11,14]. This intricate interplay between the centralization of vascular services, referral times, and patient outcomes necessitates a nuanced examination to elucidate its multifaceted impact on the CEA pathway.

Within this context, our study embarks on an exploration to discern the influence of centralized services on the timely execution of surgeries for patients referred due to symptomatic carotid artery stenosis. By delving into the interplay of factors affecting referral and procedural times, our investigation aims to offer a comprehensive understanding of the ramifications of centralization on this critical aspect of vascular care.

## Materials and methods

**Figure 1:**
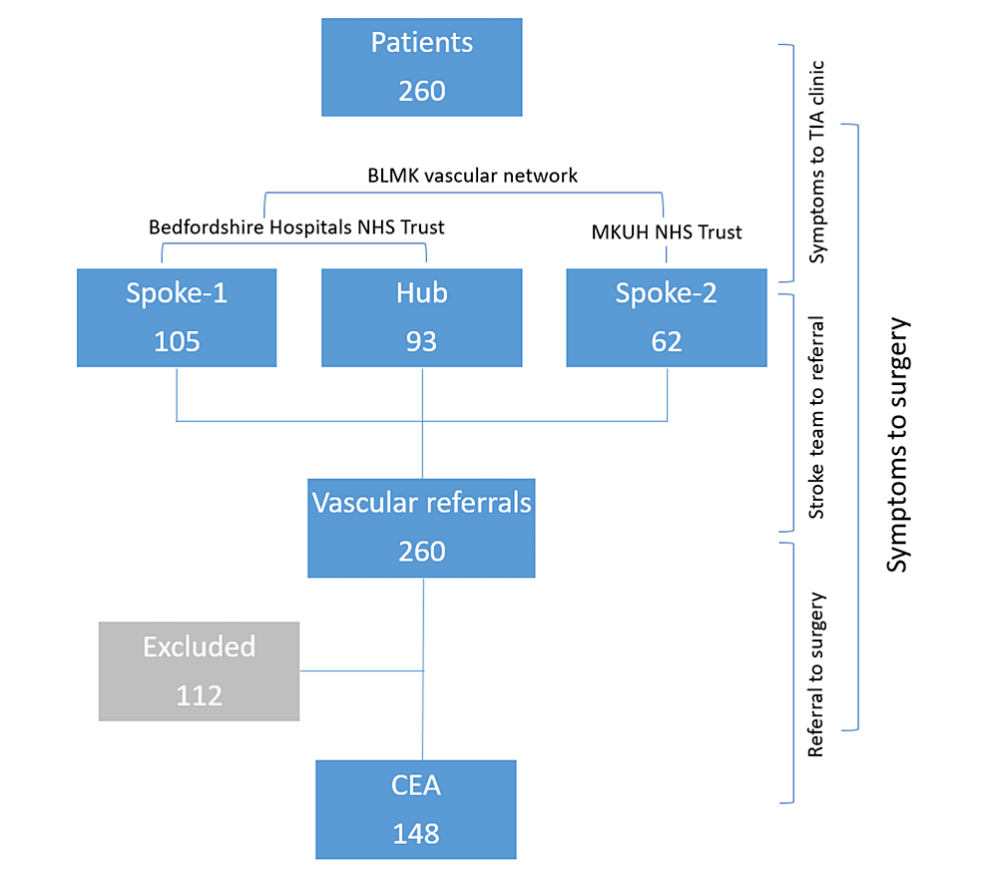
Patient selection, vascular unit structure, and timeline representation BLMK vascular network consists of Bedfordshire Hospitals NHS Foundation Trust (i.e., Bedford (Hub) and Luton and Dunstable University Hospital (Spoke-1)) and MKUH Foundation Trust (Spoke-2). The timeline assessed was symptoms to stroke team review, stroke team review to referral, and referral to surgery. The overall time was also evaluated from symptoms to surgery. BLMK, Bedfordshire, Luton, and Milton Keynes; NHS, National Health Service; MKUH, Milton Keynes University Hospital; CEA, carotid endarterectomy; TIA, transient ischemic attack

This study adopted a retrospective approach to investigate patients who underwent CEA at the Bedfordshire, Luton, and Milton Keynes Vascular (BLMK) network from January 2021 to June 2023. Inclusive criteria included patients diagnosed with symptomatic carotid stenosis who subsequently underwent CEA. Excluded from the study were patients who underwent CEA for asymptomatic stenosis, those medically unsuitable for surgery, and those considered for best medical therapy (Figure 1).

BLMK vascular network structure

The BLMK vascular network comprises three hospitals affiliated with two National Health Service (NHS) trusts. Bedfordshire Hospitals NHS Foundation Trust, consisting of two hospitals, includes Bedford Hospital, which operates as the Hub and offers 24/7 onsite vascular services with a team of seven consultants. Luton and Dunstable University Hospital, designated as Spoke-1, is staffed by three onsite vascular consultants who provide three days of weekly coverage. Milton Keynes University Hospital NHS Trust, referred to as Spoke-2, is supported by two vascular consultants.

Referral pathway

Patients presenting with an ischemic event undergo an evaluation by the stroke team. This evaluation includes a comprehensive assessment and diagnostic imaging to detect carotid artery stenosis. Subsequently, the patient is referred to the vascular team through a standardized referral system, ensuring their clinical evaluation by vascular consultants. The patient's suitability for surgical intervention is assessed following established clinical guidelines and a modified Rankin Score; the surgery is prioritized at the earliest date (Figure 1) [9,15].

Data collection and analysis

The demographic data and referral specifics were acquired from each hospital database. Demographics included age at presentation, type of event such as TIA, amaurosis fugax, non-disabling stroke (herewith termed stroke), and any other causes. The patient cohort was categorized into three groups predicated on their primary referral point within the BLMK vascular network. These patient groups are the Hub, Spoke-1, and Spoke-2. The temporal sequence of events was denominated in days. These events included the duration between the index event and the subsequent review by the stroke team, the interval from stroke team assessment to vascular referral, referral to the surgical intervention, and the overall duration spanning from the onset of symptoms to the actual surgical procedure.

Statistical analysis was conducted using Minitab software (version 21 for Windows, developed by the Pennsylvania State University). Age at presentation was expressed as the mean value with standard deviation. Gender, the type of ischemic event, laterality, referring hospital, the occurrence of a second event, and the rate of surgery performed within 14 days were reported as frequency and percentage. The presentation, stroke review, referral, and surgical intervention durations were presented as mean values in days with standard deviations.

The temporal interplay between the source of patient referral, the presenting events, and the surgical intervention's timing was assessed by Classification and Regression Tree (CART) analysis and receiver operating curve (ROC). Delays in the context of a predetermined standard cut-off value of 14 days, along with second events and referral sites, were assessed and represented by box plots. The variability of delays between the Hub and Spoke sites while scrutinizing the time element by considering the year of referral was represented with box plots. Fisher's exact and chi-square tests were utilized for 2x2 tables containing categorical data with statistical significance defined as a p-value less than 0.05.

## Results

This study included 148 patients after applying exclusion criteria. The mean age was 73.7±9 years, with 71.8% (n=107) male. Predominant clinical presentations were TIA in 67.8% (n=101) and amaurosis fugax in 16.8% (n=25). Patients were categorized by referral origin (Hub: 35.5%, n=53; Spoke-1: 45.6%, n=67; Spoke-2: 18.8%, n=28) (Table 1).

**Table 1:** Basic demographics, second events, and time to surgery The data are represented as N (number, frequency), % (percentage), and mean±SD. TIA, transient ischemic attack; CEA, carotid endarterectomy

Category	Group	Value
Age		73.7±9
Gender	Male	107 (71.8%)
	Female	41 (28.2%)
Indication	TIA	101 (67.8%)
	Amaurosis fugax	25 (16.8%)
	Stroke	21 (14.1%)
	Seizures	1 (0.6%)
Side	Left CEA	73 (49%)
	Right CEA	75 (51%)
Hospital	Hub	53 (35.5%)
	Spoke-1	67 (45.6%)
	Spoke-2	28 (18.8%)
The second event after the index event		23 (15.4%)
	Before the stroke team review	10 (6.7%)
	After the stroke team review	14 (9.4%)
Surgery within 14 days	Yes	59 (40%)
	No	89 (60%)

CEA was performed within the recommended time in 40% (n=59) of cases, while 15.4% (n=23) experienced a second ischemic event before surgical intervention. The mean time for stroke team review was 3.6±8.4 days, with 5.5±8 days of time to referral. Spoke-2 exhibited the most extended duration for this interval at 9.6±13.2 days. The total time from symptom to surgery was 25.6±24 days (Table 2).

**Table 2:** Time differences and second events comparison with Hub and Spoke sites The data are represented as N (number of days) and mean±SD. TIA, transient ischemic attack

Period category	Hub	Spoke-1	Spoke-2	Total
Symptoms to stroke team review	4.7±12.2	2.6±5.4	3.6±5.3	3.6±8.4
Stroke team to vascular referral	5.7±8	3.6±3.9	9.6±13.2	5.5±8
Vascular referral to surgery	14.5±15.3	18.3±25.8	15.8±13	16.5±20.4
Symptom to surgery	25±22.9	24.5±27	29±21	25.6±24

15.4% (n=23) experienced second ischemic events while awaiting CEA from the onset of the index event. The occurrence of these events was higher at Spoke-2 (25%, n=7) compared to Hub (17%, n=9) and Spoke-1 (10.3%, n=7). Notably, 6.7% (n=10) of patients encountered these events before their assessment by the stroke team, while 9.4% (n=14) events happened after the review; this was statistically significant, p=0.03 (Table 3).

**Table 3:** Period when a second ischemic event happened The data are represented as N (number of days) and % (percentage). A p-value of <0.05 is considered significant.

Period of second event	Event	Hub	Spoke-1	Spoke-2	p-value
Index event to surgery	Yes	9 (17%)	7 (10.3%)	7 (25%)	0.2
	No	44 (83%)	61 (89.7%)	21 (75%)	
Before stroke team review	Yes	5 (9.4%)	4 (5.9%)	1 (3.5%)	0.5
	No	48 (90.6%)	64 (94.1%)	27 (96.5%)	
After stroke team review	Yes	5 (9.4%)	3 (4.4%)	6 (21.5%)	0.03
	No	48 (90.6%)	65 (9.6%)	22 (78.5%)	

**Figure 2:**
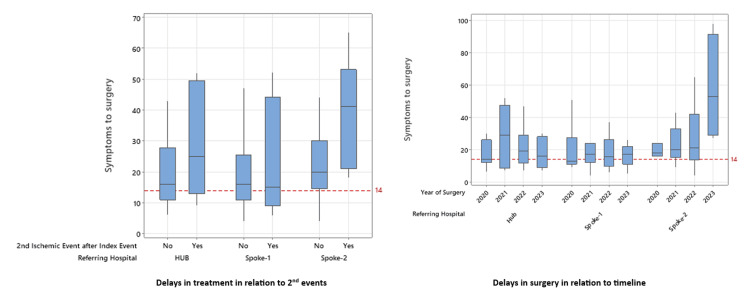
Analysis of delay in CEA The box plot (left) illustrates surgical delays concerning the occurrence of a second ischemic event and the Spoke-Hub sites. The box plot (right) depicts surgical delays in different years and their association with Spoke-Hub sites. The y-axis represents data in days, with the dashed red line denoting the recommended time frame for CEA. CEA, carotid endarterectomy

Box plot analysis revealed that a second ischemic event did not significantly affect the timing of surgery in Hub and Spoke-1. However, Spoke-2 experienced the most delays, frequently exceeding the 14-day surgical window (Figure 2). An assessment of temporal trends in delay patterns indicated that recent years witnessed more pronounced delays in Spoke-2, although no substantial differences were observed in Hub and Spoke-1 (Figure 2).

**Figure 3:**
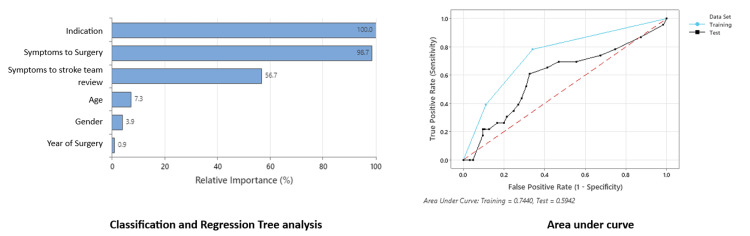
Importance of symptoms to delay of surgery Variable importance measures model improvement when splits are made on a predictor. Relative importance is defined as % improvements with respect to the top predictor.

CART analysis indicated that amaurosis fugax and stroke were the most influential factors associated with recurrent ischemic events, with 23.7% (nine patients) experiencing a second event before surgical intervention (Table 2). Notably, the duration from the index event to the stroke team review emerged as an essential contributing factor, with a relative importance of 56.7% (Figure 3). The analysis implies that patients presenting to the emergency department with subsequent delays in stroke team review or who experienced an event but did not seek timely assessment were at higher risk of experiencing a second event (Figure 3).

## Discussion

Centralization of vascular services, often organized into a "Hub and Spoke" network, has gained considerable attention in the medical community due to its potential to enhance the quality of care and improve patient outcomes [10,11,16,17]. We explore the literature on centralization, specifically focusing on CEA, the challenges in achieving timely intervention, and the implications of centralization on delivering vascular services.

Numerous studies have supported the centralization of vascular services, involving the concentration of specialized care in high-volume centers, emphasizing the relationship between case volume and outcomes [18]. Higher volume centers have demonstrated superior results, with reduced morbidity and mortality rates aligning with the broader concept that concentrating expertise and resources in specialized centers can improve patient care. However, centralization is a multifaceted process that extends beyond geographical consolidation. It encompasses creating a network comprising Hub and Spoke sites to ensure efficient patient management and resource utilization [10,11,16,17].

Clinical guidelines have underscored the importance of timely intervention for patients with symptomatic carotid artery stenosis. Specifically, guidelines recommend that CEA be performed within 14 days of the onset of symptoms to mitigate the risk of a second ischemic event, potentially resulting in a disabling stroke [9,19]. The practical feasibility of consistently achieving these targets has remained a subject of inquiry, given the complex dynamics of healthcare systems [9,20].

Recent research has shed light on the challenges associated with meeting the recommended timeline for CEA. Studies indicate that only a minority of patients, approximately 20%, undergo surgery within the critical 14-day window, suggesting that delays are pervasive [21]. These delays have been attributed to several contributing factors, including delays in TIA review, delays in diagnostic imaging, and variations in the speed of referrals [1].

Furthermore, the type of hospital or unit where the patient initially presents is a critical factor. Research has revealed that the referral source significantly influences surgical intervention timing. Notably, referrals from the stroke team, expected to expedite the process, have still resulted in substantial delays [21].

The centralization of vascular services has introduced a paradigm shift in managing vascular conditions. However, it has also raised questions about the ability of Spoke sites to uphold the standards of timely care established at Hub centers. Spoke sites may face challenges in achieving timely review and intervention, potentially due to lower oversight or variations in resource allocation [10]. One study comparing pre-centralized and post-centralized data reported an improvement in meeting the national target of 14 days, but it also noted variations in service provision post-centralization [12]. While these variations were not deemed significant, they underscore the need for ongoing monitoring and evaluation of centralization efforts.

Many post-centralization studies have focused on patient outcomes while overlooking the quality of integrated services at Spoke sites. This study recognizes the importance of understanding and addressing Spoke sites' specific challenges, as they may experience difficulties in actively coordinating referrals with the vascular team. Furthermore, the study reveals the importance of developing multi-disciplinary strategies to manage patients with reversible ischemic events across all Spoke sites.

Study limitations

Limitations of this study encompass the data concerning delays preceding the stroke team review, making it difficult to ascertain the impact of patient factors. Our study excluded the cases that did not have CEA and thus did not compare the interplay of non-surgical management and its reasoning. 

## Conclusions

Our study highlights the impact of the Hub and Spoke model on vascular surgery outcomes. A concerning trend of increasing referral delays from Spoke sites has emerged as centralization advances. This study unequivocally demonstrates the potential for these delays to disrupt the timely care delivery. Consequently, we advocate implementing a multi-disciplinary team approach in Spoke sites. This approach, promoting collaboration among healthcare professionals, offers a strategic solution to expedite patient referrals and maintain standardized, high-quality care.
